# Analysis of Electromyographic Signals from Rats' Stomaches for Detection and Classification of Motility

**DOI:** 10.3390/s8052974

**Published:** 2008-05-06

**Authors:** Laura Ivoone Garay Jiménez, Pablo Rogelio Hernández Rodríguez, Roberto Muñoz Guerrero, Emma Gloria Ramos Ramírez

**Affiliations:** 1 Básicas de Ingeniería, Unidad Interdisciplinaria de Ingeniería y Tecnologías Avanzadas, Instituto Politécnico Nacional, ave. IPN 2580, Col. La Laguna Ticomán, GAM, México D.F., México, C.P. 07340; 2 Bioelectrónica, Ingeniería Eléctrica, CINVESTAV- IPN, 2508, Col. San Pedro Zacatenco, México D.F. México, C. P. 07360; E-mail: pablo.rogeli@cinvestav.mx; 3 Biotecnología y Bioingeniería, CINVESTAV-IPN, 2508,Col. San Pedro Zacatenco, México, D.F. México, C.P. 07360; E-mail: eramos@cinvestav.mx

**Keywords:** Motility, artificial neural network, stomach contraction, ultrasound system, electromyographic signals

## Abstract

This paper presents the analysis of the electromyographic signals from rat stomachs to identify and classify contractions. The results were validated with both visual identification and an ultrasonic system to guarantee the reference. Some parameters were defined and associated to the energy of the signal in frequency domain and grouped in a **P** vector. The parameters were statistically analyzed and according to the results, an artificial neuronal network was designed to use the **P** vectors as inputs to classify the electrical signals related to the contraction conditions. A first approach classification was performed with and without contraction classes (CR and NCR), then the same database were subdivided in four classes: with induced contraction (ICR), spontaneous contraction (SCR), without contraction due a post mortem condition (PMR) or under physiological conditions (PNCR). In a two-class classifier, performance was 86%, 93% and 91% of detections for each electrogastromyografic (EGMG) signal from each of three pairs of electrodes considered. Because in the four-class classifier, enough data was not collected for the first pair, then a three-class classifier with 82% of performance was used. For the other two EGMG signals electrode pairs, performance was of 76% and 86% respectively. Based in the results, the analysis of **P** vectors could be used as a contraction detector in motility studies due to different stimuli in a rat model.

## 1. Introduction

In the literature, there is a controversy about whether the stomach of the rat really has a myoelectrical signal similar to those presented by other biological models such as humans and dogs [1-5]. Reliable knowledge about the motility of the rat stomach could be relevant because this biological model could be widely used in research, mainly in trials to determine relationships between bolus effects, some metabolic disorders or drug pharmacokinetics with stomach motility [5-8,16]. Electrical signals from biological models may be different in shape, but similar in the functions they represent. Consequently, the establishment of a relationship between electrical signals and functions of the stomach, through the motility, could be very useful for research and clinical studies.

The dog model has been widely used for mechanical and electrical analysis of the performance of the stomach [10-12]. However, experimentation with that model has become very difficult at the present time, because of several constraining regulations [13-14] and economical factors.

On the other hand, the rat has become an interesting biological model because has a stomach with mechanical and electrical similarities to the human stomach [3,6]. In this sense, studies of hormones, pH, and pharmaceutical actions have been done [6-9]. Some reports use the myoelectrical signal in the stomach of rat as an electrical pacemaker monitor [3,5,8], but the literature also recognizes that a detailed knowledge of gastric motility in rat stomachs related to electrical signals is required [1-3,5,6,16].

Nowadays, the gastric myoelectrical signal in human and dog is considered to be composed by two kinds of signals: the electrical pacemaker signal (ECA) and a stochastic signal called spikes or electrical response activity (ERA), associated to contractions. The pacemaker signal has been widely studied, whereas the ERA analysis has been rarely approached.

In this study, the rat was used as the experimental model for a gastric my electrical signal analysis in search of a parameter that could be used as a contraction identifier. By means of analysis in frequency domain of the electromyographic signals, parameters based on the segmentation of the power spectrum in fixed blocks were defined in this work. These parameters were used for identifying events with and without contractions by statistical analysis and for classifying contraction conditions performed by an artificial neural network. Based on the obtained results, this technique can be considered not only as an electrical pacemaker monitor, but also as a contraction analyzer.

## 2. Methods

### 2.1. Subjects and protocol

Nine Wistar male rats (250 g average weight) were used. Each experiment involved a rat with 24-hours fast and water *ad libitum*. Intraperitoneal anesthesia with penthobarbital (SEDALVET, Tokkyo, México) was applied. The rat was placed on a thermal bed in supine position. A laparoscopy was realized in order to expose the stomach but preserving innervation and blood irrigation. The stomach was maintained hydrated with saline solution (0.9%) during all procedures. The body temperature and the respiratory frequency were recorded for monitoring the physiological conditions. All experiments were performed following our institutional guide and regulation manual to research with animals [13, 14]. Electrogastromyographic (EGMG) signals were sampled with a frequency of 20 Hz and acquired in a personal computer-based system for off-line processing. Signals were marked when contractions were visually observed on the exposed stomach wall. These records were used as a reference for the classification of the database. The experimental protocol was divided into four procedures:

#### The first procedure

**Figure 1.**
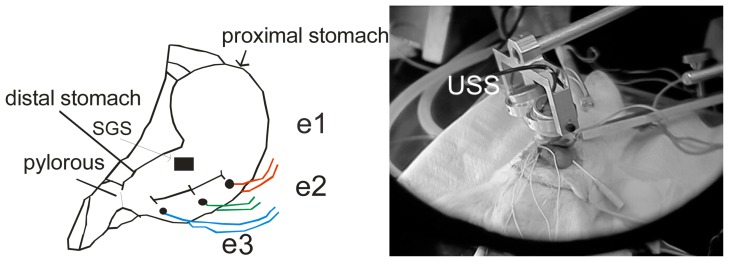
Experimental protocol design. SGS: strain gage system, USS: Ultrasonic system, *e1*, first pair of electrodes; *e2*, second pair of electrodes, and *e3*, third pair of electrodes.

Three pairs of Ag/AgCl electrodes were placed along the main curvature of the stomach. The first pair was placed at 20% of the total distance between the proximal zone and the pylorus. The second and third pairs were placed 4 mm below the previous pair (Figure 1). An ultrasonic system (USS, customized system, CINVESTAV, Mexico) was placed over the distal zone [15]. A sensor for respiratory monitoring and a rectal temperature sensor were used. At least five consecutive records, under physiological conditions, of 2.5 minutes in duration were obtained.

#### The second procedure

A system for detecting the stomach motility based on a strain gage (SGS, customized system, CINVESTAV, Mexico) was used, with the sensor placed next to the second pair. The USS was placed over the strain gage zone. The signals were consecutively recorded during at least five times under same physiological conditions.

#### The third procedure

A pylocarpine solution (Pil^®^, Sophia, Bulgaria) was applied on the surface of the stomach wall in order to obtain contractions in the distal zone. Records were obtained while motility effects of the drug were observed on the wall.

#### The fourth procedure

Overdose of pylocarpine solution was applied and respiratory failure was induced. After 20 minutes, the last records were obtained.

### 2.2. Data pre-processing and conditioning

The EGMG signals were sampled at 20 Hz and then grouped per experiment. The maximum and minimum values of magnitude were obtained for each experiment and the records were normalized from 1 to -1. Three consecutive records of 2.5 s in duration of the same experiment, obtained from each of the four procedures were used as signal sets. Then, the spectra of these sets were computed and values of the parameters were obtained. In order to compare the overall results of the nine experiments, maximum and minimum values were used for normalizing all experiments from 1 to -1.

### 2.3. Parameter selection

The frequency spectrum of the electrical signal database was obtained using the Fast Fourier Transform (FFT). When the analysis of the electrical signals in frequency domain was performed, an increment in energy was observed in a band of the spectrum, around the corresponding frequency component of the pacemaker, reported as 0.05 Hz or three contractions per minute in rats [3, 4].

After a visual inspection, the frequency changes were mainly observed from low frequencies up to 1.5 Hz. Then, a frequency subdivision of spectrum was proposed and energy contained in each band was associated to a parameter p_i_ and used for the signal analysis (Table 1).

Additionally, the mean of p_i_ parameters (mean), the Root Mean Square (RMS), the maximum magnitude (MM) in each power spectrum frequency and the frequency associated to MM (MF) were obtained. All these parameters were grouped in vectors named **P** = [p1 p2 p3 p4 p5 mean RMS MF MM]. The last two parameters represent the maximum energy concentration related to the pacemaker frequency, reported as 3 cpm (0.05 Hz) for the rat model [3, 4, 6, 7]. The generated database of parameters vectors was obtained from the spectra of EGMG signals of three electrode pairs using 50 records with contraction and 50 with no contraction at least.

### 2.4. Parameter validation

The obtained parameters were classified as with or without contractions, according to the visual detection. Because visual detection was used as a reference, a customized ultrasonic system (USS, CINVESTAV, Mexico) was also employed as a redundant contraction detector to guarantee reliable information [15].

The information was divided into two classes for analysis: with (CR) and without (NCR) contractions. In turn, each class was divided into two subclasses: contractions pharmacologically induced (ICR) and spontaneous (SCR) for the subclasses of the first class. In the second class, subclasses were assigned for under physiological conditions (PNCR) and for post-mortem records (PMR). The aforementioned is summarized in Table 2, as well as the number of vectors for each class.

### 2.5. Statistical analysis

Each parameter was analyzed with ANOVA for repeated measurements, looking for a single parameter which could be used as contraction detector. Based on the statistical analysis results, the first seven parameters of the **P** vector were used for classifying the signals employing an artificial neural network. Because maximum magnitude (MM) and its frequency (MF) represent the pacemaker presence in the frequency domain [3], but its effect is included in p2, the last two parameters wer proposed for pacemaker analysis and they were not considered in the classification inputs.

### 2.6. Classifier

The classification was performed with a backpropagation artificial neural network formed by a input layer with seven inputs, one hidden layer with four neurons, and an output layer with fou neurons. Hyperbolic tangent sigmoid and linear transfer functions were used for processing in hidde and output layers respectively. As a first approach, this structure was used for classifying the case related to with and without contractions (CR and NCR). Then, it was used for identifying four different subclasses: ICR, SCR, PNCR, and PMR.

The test set was the 25% of the total cases for each electrode pair, randomly selected. Remain cases were used as the training set. Weight and bias of each layer were initialized with the Nguyen-Widrow method and the network was trained with the Levenberg-Marquardt backpropagation method. The obtained output vector was matched to target vectors with minimal mean squared error (MSE). The maximum iteration number and the maximum error value were selected for each electrode pair according a previous analysis of the training performance. Processing was performed with the neural network toolbox in Matlab ® version 7 (The Mathworks Inc., USA).

The target vectors used a specific combination of the output neurons for each class, for example in two classes case, the target vectors were T1 = [-1 -1 1 1] for CR class and T2 = [1 1 -1 -1] for NCR class.

For each vector of the testing set, the obtained value of each neuron was adjusted to the two possible values, following the next rule: If the value was more than zero, then the adjusted value was 1, otherwise -1.

Then each adjusted neuron value was compared with the expected target value, and if the four output neurons values were the same, a correct classification of the vector was performed.

After selecting a test set, training and classification was performed. To demonstrate non over fitting, a k-fold cross validation with k=10, was performed. Additionally, for evaluating the effect of the selection of the initial weights and bias values, the training and testing were repeated three times with the same set (r=3).

The success of each classifier was obtained from the correct classification (CC) of the 25% of total **P** vectors for each electrode pair, repeated three times (r). For example, *e1* has a 24 total number of **P** vectors and the 25% are 6 vectors, so in this case TC1=3 x 6 =18. Summary of the TC1 for each pair is presented in table 3 as well as the results of performance for each classifier.

Finally, success of the structure of the neural network used as a classifier is evaluated. All k times validation results are presented in table 4 and the total test cases considered was defined as:

TC= k * TC1.

## 3. Results and Discussion

### 3.1. Statistical analysis results

The statistical analysis of the **P** vector was performed for the three pairs of recording electrodes. With the first pair *e1*, no sufficient data could be obtained in the first procedure related to spontaneous contractions. Then, this case was not considered in the four-class detection.

**Figure 3.**
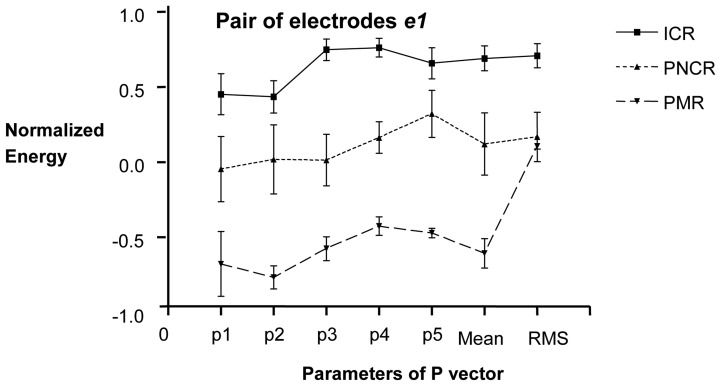
Mean + SEM of **P** vector of the EGMG from the first pair of electrodes *e1*. Energy data of ICR: induced contraction records, PNCR: physiological non contraction records, PMR: post mortem records were normalized. The x-axis shows the elements of the **P** vector.

For the pair of electrodes *e1*, the parameters from the four classes could be statistically differentiated except RMS because of the large magnitude of its standard error of the mean (SEM), shown in Figure 3. However, considering the **P** vector as a set of information, it could be used as a contraction condition identifier.

**Figure 4.**
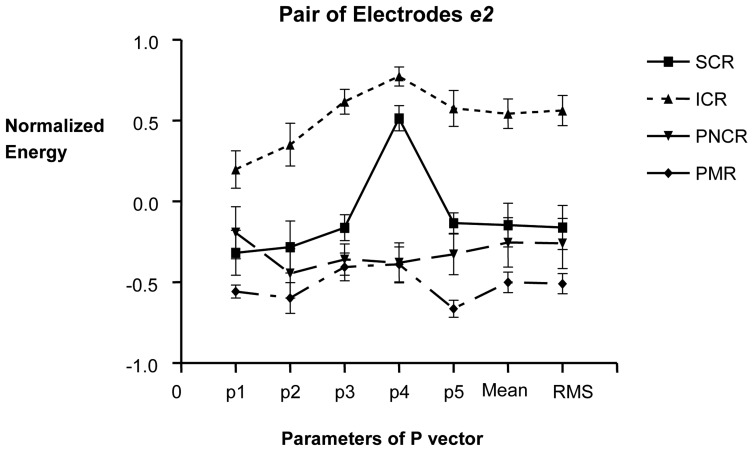
Mean + SEM of **P** vector of the EGMG from the pair of electrodes *e2*. Energy data of ICR: induced contraction records, SCR: spontaneous contraction records, PNCR: physiological non contraction records, PMR: post mortem records were normalized. The x-axis shows the elements of the **P** vector.

In the second pair of electrodes *e2*, the parameter p4 presented the biggest difference between contraction conditions (Figure 4). In this pair, the parameter magnitudes were higher for records with than without contractions. Again, considering the **P** vector, contraction condition could be identified.

**Figure 5.**
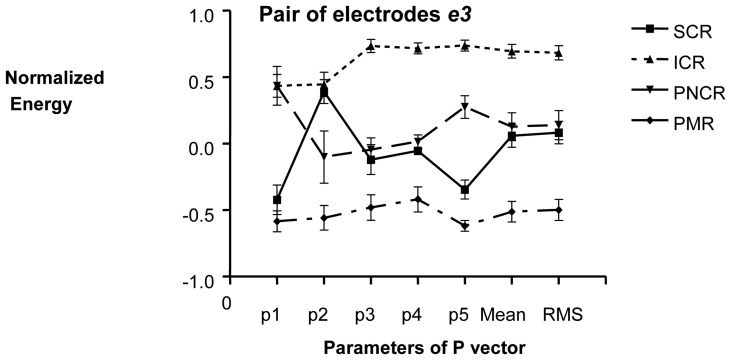
Mean + SEM of **P** vector of the EGMG from the pair of electrodes *e3*. Energy data of ICR: induced contraction records, SCR: spontaneous contraction records, PNCR: physiological non contraction records, PMR: post mortem records were normalized. The x-axis shows the elements of the **P** vector.

In the pair of electrodes *e3*, the parameter p5 presented the largest differences among contraction conditions (Figure 5). In this case, contraction conditions could be statistically identified for the four classes. The **P** vector could also be used for identifying the contraction conditions. Moreover, it was found that p2 parameter in two of the three electrode pairs could be used as a good pacemaker detector. This finding was expected because p2 (signals between 0.04-0.1Hz or 2.4-6 cpm), is closely related to the reported pacemaker frequency (3 cpm) [1,3,4].

### 3.2. Classifier

As mentioned above, an artificial neural network was proposed to be used as a classifier. The output layer of the neural network had four neurons ranged from 1 to -1. The target vector was assigned to each class for each classifier.

The maximum number of epochs was established when the maximum error goal was obtained at least in five trainings or when a larger number of epochs did not represent a less error in the training results. Training considered all the 10-fold cross validation sets.

In training for each electrode pairs (e1, e2 and e3), maximum number of epochs were from 1200 to 1700 with a performance goal from 0.05 to 0.1.

The first approach was to classify with or without contraction classes (CR or NCR). The results of each classifier are presented in Table 3 and the overall results from the k classifiers are shown in Table 4. The classification was possible in at least 86% of the cases in all the electrode pairs (Table 4).

The second approach was used with the four classes ICR, SCR, PNCR, and PMR. In this case, the overall identification of classes for e2 and e3 pairs of electrodes was 76% and 87% respectively. For the first pair of electrodes (*e1*), not enough data were obtained, then, this case was not considered.

For the pair of electrodes *e1*, three classes were analyzed and an overall performance of 82% was obtained.

Because a successful classification was defined when the four neurons values were the same as the target value, and it was repeated three times for each k classifier, results were summarized per classifier. These results are presented in Table 3 and the overall performance of the neuronal network structure in Table 4.

### 3.2. Discussion

The use of the ultrasound system as a redundant contraction system to complement the visual identification ensured the initial identification of the classes as a reference for the evaluation of the contraction monitor based in the analysis of the **P** vector proposed in this work.

The **P** vector was initially proposed with nine elements because inclusion of the maximum magnitude peak of the density spectrum (MM), which is associated with the pacemaker frequency (MF) has been traditionally included in monitor detectors. In this study, the presence of the electrical pacemaker was observed as well as an increase in the magnitude of the spectral density due to the presence of contractions, as it has been reported by several authors [1,3,4,7]. Moreover, it was observed that the pacemaker frequency magnitude was significant in all pairs of electrodes when the rat model was under physiological condition. Once a*post mortem* condition was induced, the MM (maximum magnitude) parameter decreased until it was not differentiated from the rest of the spectrum components. The effectiveness of those two parameters as pacemaker detectors was comparable with the p2 parameter capacity to recognize the presence of the pacemaker frequency (MF). Then, given the good results to detect the pacemaker activity and to identify contractions by using the **P** vector, the parameters MF and MM were not considered in the signal processing anymore.

## 4. Conclusions

The EGMG signal was studied in the distal stomach of rats in order to look for parameters that could be used as contraction identifiers. According to the results, the parameter **P** vector could satisfactorily fulfill that purpose. Analyzing the **P** vector, identification of contractions from the EGMG signal of rats as the biological model was systematically possible

The use of an artificial neural network as a classifier makes possible the detection of contractions associating the conditions they were produced. This technique could identify the spontaneous or pharmacological induced contractions as well as the absence of them due to the lack of any electrical or mechanical activity, after a minimum pre processing of the signal in order to obtain the spectrum and maximum and minimum of the signal set.

This contraction detector could be used to study the effects on motility of the stomach related to different stimuli in a rat model.

## Figures and Tables

**Figure 2.**
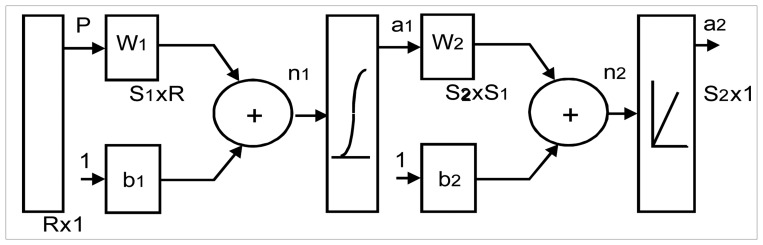
Artificial Neural Network proposed. **P** is the input vector, Rx1 is the dimension of the **P** vector, **W**_1_ and **W**_2_ are the initial weights for each neuron; b_1_ and b_2_ are the bias for each neuron; n_1_ was the output of the first layer. S_1_ is the number of input neurons and S_2_ is number of output neurons; **a**_1_ and **a**_2_ are the results of the hidden and output layers.

**Table 1.** Energy of frequency bands was quantified and it was proposed as a parameter for classification of electrogastromyographic signal.

Parameter **Pi**	Band of the spectrum considered in Hertz (Hz)	Band of the spectrum considered in contractions per minute (cpm).
p1	0.01 – 0.04	0.6 – 2.4
p2	0.04 – 0.1	2.4 – 6
p3	0.1 – 0.5	6 – 30
p4	0.5 – 1	30 – 60
p5	1 – 1.5	60 – 90

**Table 2.** Summary of the total vectors (TC) obtained from the overall EGMG database. Each set used for quantifying the spectrum of the EGMG signals of three electrode pairs that were obtained simultaneously from the stomach tissue. CR stands for contraction condition and NCR is non contraction condition.

Electrode Pair	Total parameter vector (TC)	Vectors classified as CR		Vectors classified as NCR	

		ICR	SCR	PNCR	PMR
e1	24	14	---	5	5
e2	59	25	16	12	6
e3	57	25	13	7	12

**Table 3.** Accuracy results of each classifier are presented as the ratio between the correct classification value (CC) and the total tested vectors (TC1).

2-class classifiers											

K	1	2	3	4	5	6	7	8	9	10	*TC1*

Electrode pairs											

*e1*	0.83	0.83	0.94	0.88	0.61	0.83	0.77	1	0.88	1	*18*
*e2*	1	0.91	0.97	0.86	0.93	0.93	0.97	0.93	0.88	0.97	*45*
*e3*	0.9	0.9	0.74	0.93	0.98	1	1	0.9	0.86	0.95	*42*

3-class classifiers											

*e1*	0.83	0.83	0.67	0.94	0.94	0.83	0.72	0.72	0.94	0.72	*18*

4-class classifiers											

*e2*	0.73	0.71	0.84	0.71	0.71	0.86	0.86	0.82	0.64	0.71	*45*
*e3*	0.93	0.93	0.83	0.81	0.98	0.9	0.83	0.93	0.83	0.76	*42*

**Table 4.** Overall results of the neural network structure are presented as the mean of successful classification and its standard deviation over total tested vectors evaluated (TC). CR stands for contraction class, NCR for non contraction class, ICR for induced contraction class, CR for spontaneous contraction class, PNCR for physiological non contraction class and PMR for post mortem non contraction class.

Number of classes	Classes	EGMG, pair ***e1***		EGMG, Pair ***e2***		EGMG, pair ***e3***	
		CC	TC	CC	TC	CC	TC
4	ICR, SCR, PNCR, PMR.	---	---	0.76 ±0.01	450	0.87 ± 0.11	420
3	ICR, SCR, PMR	0.82 ±0.08	180	---	----	---	---
2	CR vs. NCR	0.86 ±0.14	180	0.93 ±0.077	450	0.91 ±0.14	420
